# Supplementary material for: Collagen and actin network mediate antiviral immunity against Orsay virus in C. elegans intestinal cells
Source: PLoS Pathog. 2024 Jan 8;20(1):e1011366. doi: 10.1371/journal.ppat.1011366 (PMC10798621; doi:10.1371/journal.ppat.1011366)
Supplement: S1 Fig — (DOCX) [file ppat.1011366.s001.docx]

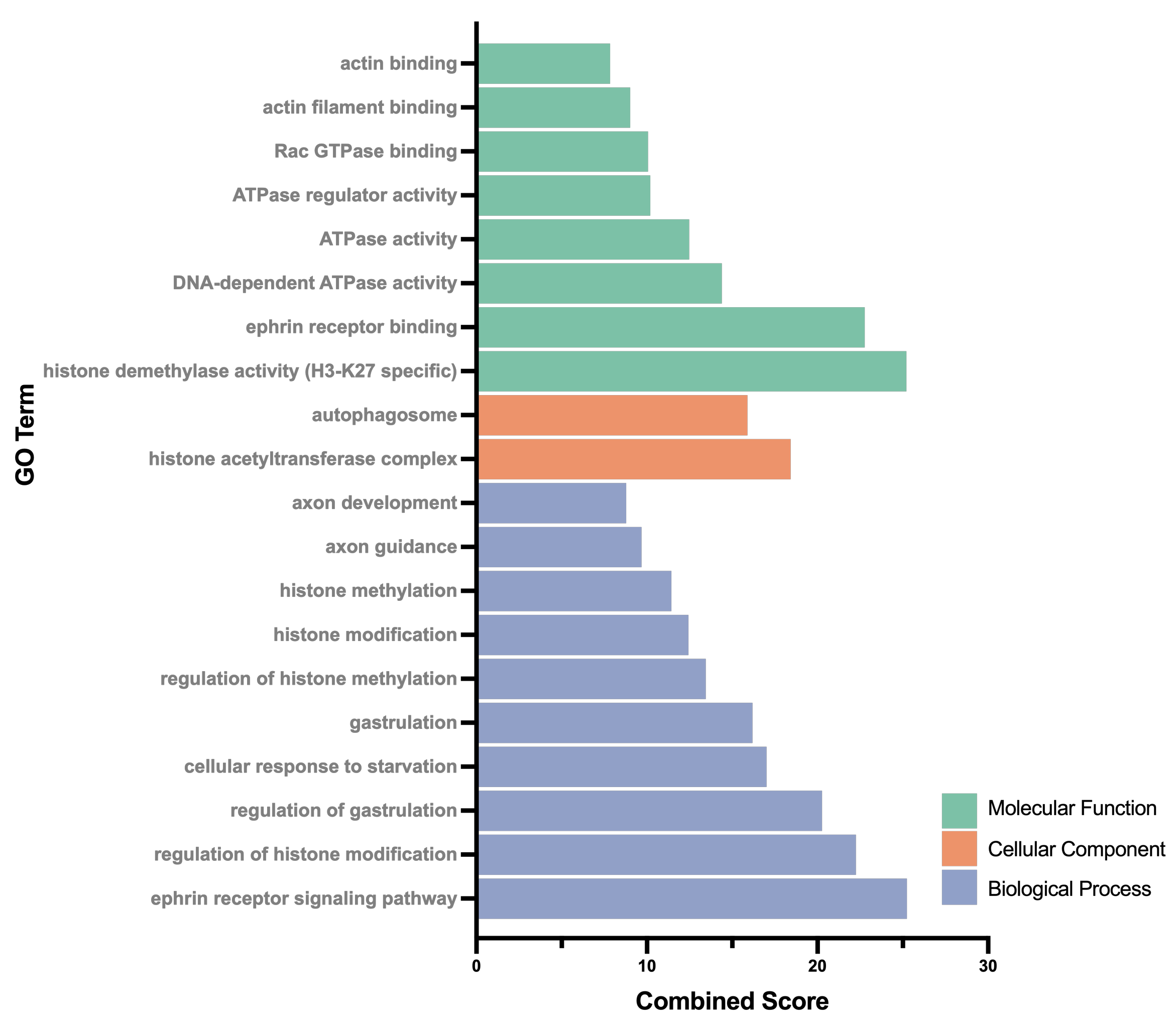


**Figure S1. Gene Ontology (GO) term enrichment analysis of the 106 potential antivirus genes.** Significant enriched GO terms in each category are shown (p< 0.05).
