# Supplementary material for: Collagen and actin network mediate antiviral immunity against Orsay virus in C. elegans intestinal cells
Source: PLoS Pathog. 2024 Jan 8;20(1):e1011366. doi: 10.1371/journal.ppat.1011366 (PMC10798621; doi:10.1371/journal.ppat.1011366)
Supplement: S2 Fig — (DOCX) [file ppat.1011366.s002.docx]

**Figure S2. Viral load detected by qRT-PCR in col-51 and N2 worms during Orsay infection.** The qRT-PCR data was set to 1 for N2 worms at 24 hpi. At least 9 independent biological repeats with 3 technical replicates were included in each experiment. p<0.001, Student’s t-test.
