# Supplementary material for: Collagen and actin network mediate antiviral immunity against Orsay virus in C. elegans intestinal cells
Source: PLoS Pathog. 2024 Jan 8;20(1):e1011366. doi: 10.1371/journal.ppat.1011366 (PMC10798621; doi:10.1371/journal.ppat.1011366)
Supplement: S3 Fig — (DOCX) [file ppat.1011366.s003.docx]

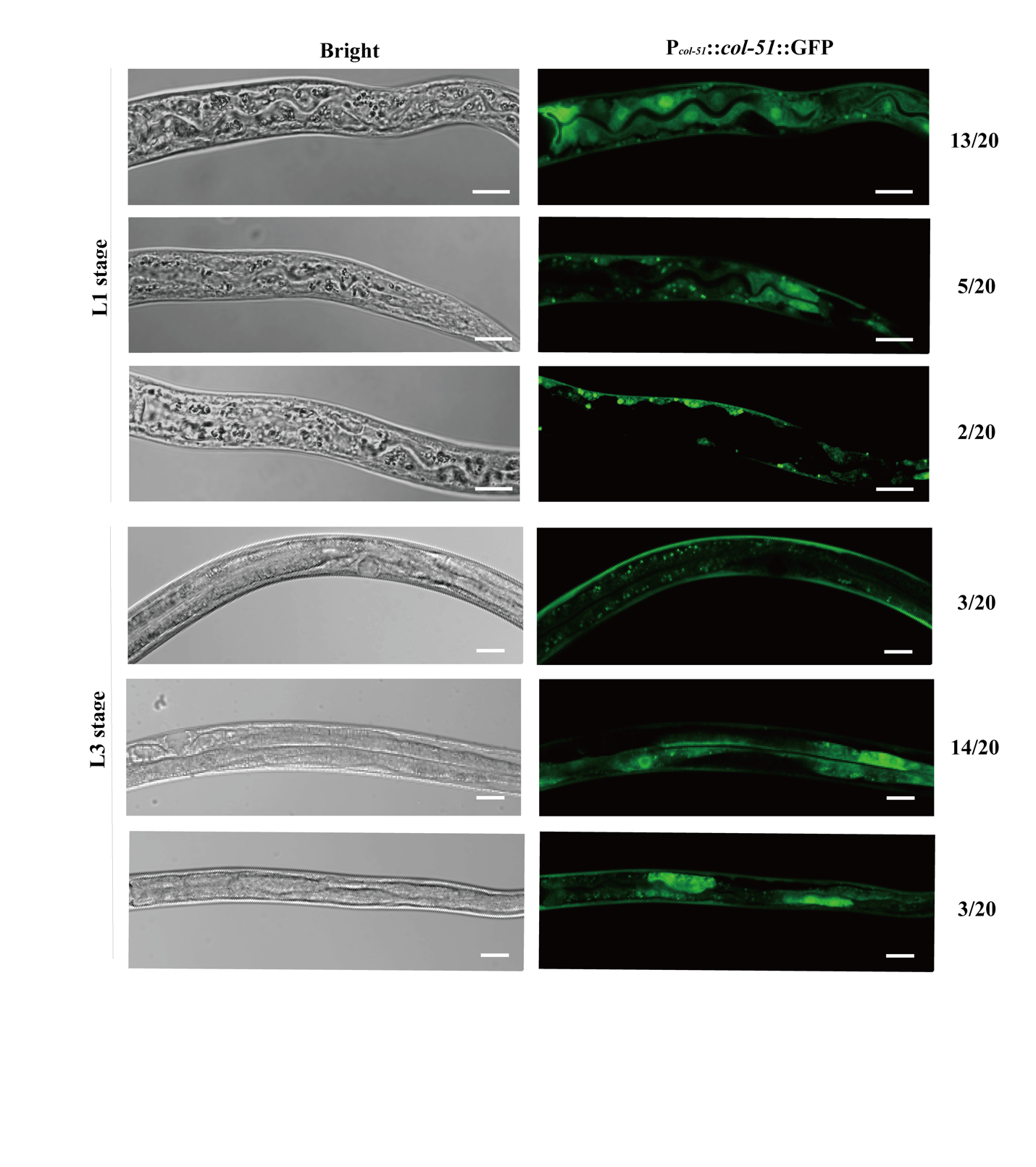


**Figure S3. Expression pattern of the *col-51* gene**. *col-51* expression was observed in both intestine and epithelium. In worms at L1 to L4 stages, three expression patterns could be detected: whole intestine, part of intestine, or epithelium. Bar: 20 μm. The numbers on the right indicate the number of worms (out of a total of 20) associated with each expression pattern.
